# How Are Sleep, Settle, and Crying Behaviors in 2‐Month‐Olds Related to Concurrent Family Factors and Later Development?

**DOI:** 10.1111/desc.70126

**Published:** 2026-01-05

**Authors:** Charlotte Viktorsson, Irzam Hardiansyah, Amelia Juslin, Terje Falck‐Ytter

**Affiliations:** ^1^ Development and Neurodiversity Lab Department of Psychology Uppsala University Uppsala Sweden; ^2^ Center of Neurodevelopmental Disorders (KIND) Division of Neuropsychiatry Department of Women's and Children's Health Karolinska Institutet Stockholm Sweden

## Abstract

Sleep, settle, and crying behaviors represent basic regulatory functions in early infancy, yet little is known about the factors that influence these behaviors and their relationship to later development in the general population. In this study, we assessed a sample of 362 infants, measuring parent‐rated number of wakeups per night, time to settle, and crying duration at 2 months of corrected age (range 27–99 days), along with various background variables. We also measured several aspects of the infants’ later development at 14 and 24 months. Age (corrected) at the first assessment showed a significant association with number of wakeups per night (*β* = –0.212, *p* < 0.001) and crying duration (*β* = –0.154, *p* = 0.012). Family income was a significant predictor of crying duration (*β* = –0.128, *p* = 0.018) and time to settle (*β* = –0.147, *p* = 0.011). Sleep, settle, and crying behaviors at 2 months were not significantly associated with parent‐rated language comprehension or socio‐communicative abilities at 14 months, nor with vocabulary, autistic traits, or hyperactivity at 24 months. While some sleep and settle behaviors in the first few months can be challenging for caregivers, our findings suggest that they are not indicators of atypical development in early childhood in the general population.

**Summary:**

- In a sample of more than 350 infants, we found that higher family income was associated with shorter time until settled and shorter crying duration at 2 months of age.
- Sleep, settle, and crying behaviors at 2 months did not show statistically significant associations with language development, hyperactivity, or autistic traits in toddlerhood.

## 1 Introduction

Sleep is a vital regulatory function that undergoes several developmental transitions as we age, reflecting changes in neural activity across multiple regions of the brain (Kohyama 1998). In newborns, the circadian rhythm is not yet established, meaning that sleep can occur as easily during the day as during the night. In most infants, a circadian rhythm based on a day‐night cycle develops at around 4 months of age (Heraghty et al. 2008), leading to less daytime sleep and an increased sleep duration during the night (Figueiredo et al. 2016). This transition is believed to be dependent on the development of forebrain circuits and the strengthening of forebrain connections with the brainstem (Blumberg et al. 2014). Since the maturation of day‐ and nighttime sleep changes throughout development, it is important to study sleep and settle behaviors at multiple time points in early childhood. In this study, settle behaviors or settle ability refers to the time it takes for an infant to become calm and ready to sleep. This construct differs slightly from sleep onset latency, which specifically describes the duration between attempting to sleep and the onset of sleep itself.

While the factors that influence sleep and settle behaviors in early infancy are not fully understood, existing evidence suggests that these behaviors may be shaped through a complex interplay of different influences, which can be viewed within a socio‐ecological framework. At the individual level, biological sex appears to be important, as girls have been found to exhibit longer sleep onset latency than boys at 3 months and longer daytime sleep at both 3 and 8 months, as well as fewer night awakenings at 3 months (Paavonen et al. 2020). At the interpersonal or family level, parental practices and routines may impact early sleep, as a recent twin study (using partly the same sample as the current study) found that shared environment accounted for 61% of the variance in night awakenings at 2 months of age (Viktorsson et al. 2025). One such parental practice is regular light‐off times in the home, which has been associated with longer nighttime sleep duration in 1‐month‐old infants (Iwata et al. 2017). At the community or environmental level, differences in nighttime sleep duration in 1‐month‐olds has been associated with seasonal changes (Iwata et al. 2017). At 1.5–3 months of age, a positive association between sleep quality and light exposure during the day has been found (Harrison 2004), although the study did not differentiate between natural and artificial light. Finally, at the societal and structural level, socioeconomic status seems to play a significant role. Lower socioeconomic status partly explains racial/ethnic differences in sleep duration at 6 months (Ash et al. 2019), and long‐term trajectories show that family adversity and lower maternal socioeconomic status during pregnancy are related to consistently shorter sleep duration and later bedtime (Manitsa et al. 2024). Taken together, these findings illustrate that infant sleep is not determined by any single factor, but rather seems to emerge from the interaction of biological, familial, environmental, and societal influences.

A growing empirical literature links early sleep to later developmental outcomes. For example, one study examining sleep from 1 week to 6 months of age in infants with and without elevated likelihood of autism found that infants with later autism (regardless of likelihood) exhibited significantly shorter 24‐h sleep duration than infants without later diagnosis (Foster et al. 2023). It has also been found that 14‐month‐olds with elevated likelihood of autism and later diagnosis exhibit shorter sleep duration, more night awakenings and more sleep onset problems than typically developing infants (Begum‐Ali et al. 2023). Shorter sleep durations between 6 and 18 months are also linked to ADHD traits at 3 years of age, an association that is not mediated by temperament (Stott et al. 2023) At 3–4 years of age, sleep problems are associated with concurrent anxiety, conduct problems and hyperactivity, and also predict these difficulties at 7 years of age (Gregory et al. 2004). Beyond socio‐emotional outcomes, there seems to be a link between early sleep and later language development. For example, higher nighttime sleep percentage at 6 months predicts receptive language at 24 months (Foster et al. 2023), and optimal sleep at 8 months has been linked to higher language development at 14 months (Hernandez‐Reif and Gungordu 2022).

Several mechanisms may account for these associations. For example, sleep is associated with brain maturation and synaptic reorganization (Pittner et al. 2023), which are important processes in early brain development. Infant sleep has also been linked to memory consolidation (Friedrich et al. 2020; Souabni et al. 2025) and visual attention (Hasshim et al. 2022). It is possible that a lack of sleep in early infancy may increase vulnerability to later cognitive and socio‐emotional difficulties through these mechanisms. At the family level, long‐term difficulties with infant sleep may strain parent‐child relationships and reduce parental well‐being.

Taken together, earlier studies point to broad associations between infant sleep and multiple aspects of later development. However, it remains unclear whether sleep represents a specific predictor of developmental outcomes or whether sleep is part of a larger constellation of traits that predicts later development. Likewise, the outcomes associated with early sleep difficulties appear wide‐ranging, raising the question of whether sleep is linked to development in a domain‐general way rather than predicting specific difficulties. In fact, sleep problems are increasingly discussed as a potential transdiagnostic marker, given the associations with a broad spectrum of developmental and psychiatric difficulties rather than with any single condition (e.g., Harvey et al. 2011). Addressing these issues of specificity, both in terms of predictors and outcomes, is critical for understanding the role of sleep in early development.

Developmental changes in sleep and settle behaviors are paralleled by a decrease in crying until around 9 months of age (McGlaughlin and Grayson 2001; St James‐Roberts and Plewis 1996). In contrast, the level of fussiness (i.e., the baby being unsettled, irritable, or fractious) is rather stable in this period (St James‐Roberts and Plewis 1996). The scarce research on what factors influence early crying suggests that both infant and prenatal factors are important. For example, temperament has been linked to daily crying duration in infancy (Barr et al. 1989), and stress and emotional problems during pregnancy have been found to increase the likelihood of the infant crying excessively at 3–6 months of age (van der Wal et al. 2007). At 2 months of age, crying duration is largely influenced by genetics (heritability estimates of 0.44–0.48), with a moderate influence of shared environment (0.34) in relation to crying duration in the evening (Viktorsson et al. 2025). There is also emerging evidence of a link between early crying and later developmental outcomes. A recent meta‐analysis found excessive crying in infancy (mean age 2.6 months) to be related to externalizing and ADHD‐related difficulties in childhood (Hemmi et al. 2011), and fussiness at 6 months has been found to be associated with ADHD traits at 3 and 5 years of age (Stott et al. 2023). One retrospective study indicates a higher rate of persistent crying in infants with later autism than in typically developing infants (Bag et al. 2018), and already at 1 month of age the cries of infants with later autism has been rated as less typical than the cries of children without later diagnosis (English et al. 2019). Polygenic score for autism has been positively associated to the duration of crying in the evening at 2 months of age (Viktorsson et al. 2025), while no associations was found in relation to crying duration during the day‐ or night‐time. Beyond developmental outcomes, excessive crying can have relational consequences, leading to negative effects on bonding and parental perception of the child (Oldbury and Adams 2015).

Research has informed us about the importance of sleep, alongside its distinct developmental trajectory during the first year of life. While some factors have been found to influence sleep and settle behaviors, much is still unknown about what impacts these behaviors in the first few months of life. Similarly, while crying often decreases as sleep and settle ability improve, our understanding of the mechanisms underlying early crying remains limited. In addition, little is known about how these behaviors relate to later development in a general population sample, and whether potential links are domain‐general or predictive of specific difficulties. Here, we examined potential biological and environmental factors influencing night awakenings, settle ability, and crying duration at 2 months of age, and the potential association between these behaviors and socio‐communicative abilities and language comprehension at 14 months, as well as autistic traits, repetitive behavior, hyperactivity, and vocabulary at 24 months. While we generally expected that the intrinsic and extrinsic factors included in this study could influence sleep and settle behaviors at 2 months of age, we had no directional hypotheses regarding these associations, due to the general lack of research at this early age. As for the associations with later development, based on earlier research (Foster et al. 2023; Hernandez‐Reif and Gungordu 2022) we hypothesized that more frequent night awakenings and longer time until the infant settles at 2 months would be associated with lower language comprehension at 14 months and smaller vocabulary at 24 months, but we have no directional hypothesis regarding crying duration. While some studies have found a link between sleep in toddlerhood and later autism diagnosis and hyperactivity (Begum‐Ali et al. 2023; Gregory et al. 2004), there is a lack of research on the link between early regulatory behaviors and later development in the general population. Therefore, we had no directional hypothesis regarding the association between early sleep and settle behaviors and later autistic traits, socio‐communicative abilities, repetitive behavior, and hyperactivity. Research aims and hypotheses were preregistered at OSF (https://osf.io/865wn/).

## 2 Methods

### 2.1 Participants

The sample consisted of same‐sex Swedish twins, who are a part of a longitudinal twin study called Gut‐2‐Twin. The participants were recruited via letters sent to families identified via the Swedish Population register, targeting families throughout all of Sweden. In total, 1925 families were invited, and 510 ultimately participated. Parent‐rated questionnaires were administered online at 2, 5, 14, 24, and 36 months. In addition, stool samples were collected as part of a larger aim to study gut microbiota, but no on‐site visits were required. Informed consent was obtained from the parents of all the children who participated. The study was approved by the regional ethics board in Stockholm (2020‐03226) and was conducted in accordance with the Declaration of Helsinki.

Exclusion criteria for the Gut‐2‐Twin study were diagnosis of epilepsy, known presence of genetic syndrome related to autism, uncorrected vision or hearing impairment, opposite‐sex twin pairs, very premature birth (prior to week 34), presence of developmental or medical condition likely to affect brain development (e.g., Cerebral Palsy), birthweight below 1.5 kg, and infants where none of the biological parents were involved in the infant's care. After data collection, some participants were subsequently found not to fulfill the above criteria and were therefore excluded due to low birthweight, anomaly in MR/CT scan, hearing impairment, cerebral palsy, seizures, and meningitis (*n* =  8 infants). In addition, 9 infants were excluded due to lack of information about zygosity. For this analysis, we also excluded participants due to twin‐to‐twin transfusion syndrome (*n* = 12 pairs of twins).

The current analysis included data from the assessments at 2, 14, and 24 months. While infants born earlier than gestational week 34 were excluded from the study, many twins are born prematurely (<37 weeks of gestation), and it is recommended to use corrected age rather than chronological age for preterm children (e.g., Gould et al. 2021). Therefore, the age at 2 months was corrected, which was calculated by subtracting the number of days between a full‐term pregnancy (39 weeks) and their gestational age from their chronological age. After the age had been corrected, we excluded participants that were either 40 days younger or older than 2 months (60 days) at the time of assessment. Due to these criteria, 69 infants were excluded from the 2‐month assessment (all due to being too old). There were no statistically significant differences between included and excluded infants regarding sex (*p* = 0.464), birthweight (*p* = 0.951), gestational age (*p* = 0.261), geographical location (*p* = 0.061), daylight exposure (*p* = 0.649), family income (*p* = 0.090), maternal age (*p* = 0.123), or paternal age (*p* = 0.307).

After exclusions, 721 participants provided data at the 2‐month assessment. As data from twins do not reflect independent observations, we included only twin 1 from each pair (categorization of twin 1 and twin 2 in each pair was based on the alphabetical order of their names). The final sample consisted of 362 infants, of which 192 (53%) were female.

### 2.2 Sleep, Settle, and Crying Measures

An online Swedish version of the Sleep and Settle Questionnaire (SSQ; Matthey 2001) was used to measure sleep, settle, and crying behaviors at 2 months of age. This is a 34‐item parent‐report questionnaire that assesses infant sleep and settling behavior. We extracted a subset of items that we combined into three scales: number of night awakenings, time until settled, and duration of crying (all variables are reported as averages over the previous week). Wakeups per night was measured using the question: “During the past week, how often on average did your child wake up at night?” Time until settled was measured using the question: “During the past week, how long did it take on average for you to get your child to settle down when it was time to sleep?” Crying duration was measured using the question: “During the past week, how long on average did your child cry during the day/evening/night?” Night awakenings was reported as the number of times per night the infant wakes up, the rest of the variables were reported as durations. Time until settled and duration of crying were reported separately for day, evening, and night. The SSQ defines day as 5 a.m.–6 p.m., evening as 6 p.m.–10 p.m., and night as 10 p.m.–5 a.m. For the primary analyses, we used the mean value of all timepoints for the settle and crying variables.

Durations were transformed to minutes and if parents reported a range (e.g., 10–30 min), the mean value was used. If it was unclear which unit was used, the response was excluded from the analysis. Answers such as “a couple of minutes” or “a few minutes” were transformed to 2 min.

While the SSQ also contained items on sleep duration, it was clear during preprocessing that caregivers did not interpret these items in a consistent manner (i.e., some reported total sleep duration and some reported duration of single sleep periods, making it impossible to discern the actual sleep duration and to create a comparable variable). We, therefore, decided to exclude all items covering sleep duration.

There were no statistically significant differences between twin 1 and twin 2 regarding wakeups per night (*t*(703) = 0.685, *p* = 0.493), time until settled (*t*(713) = 0.931, *p* = 0.352), or crying duration (*t*(711) = –0.660, *p* = 0.510).

### 2.3 Concurrent and Background Measures

We also included data on a number of intrinsic and extrinsic factors that may influence sleep at 2 months of age. These variables included age, sex, gestational age, birthweight, socio‐economic status, maternal age, paternal age, geographical location, and approximate daylight exposure at the 2‐month assessment. We chose to study daylight exposure because of the large fluctuations in daylight in Sweden throughout the year. Daylight exposure was calculated based on the geographical location of the family, consisting of the first number in the postal code (ranging from 1 to 9) of the family's home address. These postal codes were divided into four large areas covering Sweden, and daylight was calculated based on a city in the middle of each area, the 15th of each month. Daylight exposure (defined as the duration in minutes that the sun is over the horizon during a 24‐h period) was then calculated for each infant at time of assessment, based on which month the parents filled in the questionnaire and which area of Sweden they resided in. Geographical region was defined as the four large areas based on postal codes mentioned above, and ranged from 1 (furthest south) to 4 (furthest north). Descriptive statistics of the above‐mentioned measures can be found in Table 1 (see Supporting Information S1 for distributional plots).

**TABLE 1:** Descriptive statistics of background measures.

	Mean (SD) [Min; Max]	Skewness	Kurtosis
Age at 2‐month assessment^a^ (days)	66.79 (15.39) [27; 99]	−0.05	−0.47
Birthweight (gram)	2702.16 (380.41) [1700; 3900]	0.07	0.10
Gestational age (weeks)	36.68 (1.13) [34; 39]	−0.59	−0.22
Family income^b^	6.04 (2.26) [1; 10]	−0.12	−0.68
Maternal age (years)	32.15 (4.23) [24; 45]	0.33	−0.19
Paternal age (years)	33.93 (5.57) [23; 59]	1.14	2.65
Geographical region	1.76 (0.82) [1; 4]	1.08	0.92
Daylight exposure at 2‐month assessment (minutes)	728.12 (238.70) [172; 1440]	0.04	−0.93

^a^ Corrected age, calculated as the difference between full term (39 weeks) and gestational age, subtracted from chronological age.

^b^ Family income per month. Scale 1–10 where 1 = <20 K, 2 = 20–30 K, 3 = 30–40 K, 4 = 40–50 K, 5 = 50–60 K, 6 = 60–70 K, 7 = 70–80 K, 8 = 80–90 K, 9 = 90–100 K, and 10 = >100 K (SEK).

### 2.4 Follow‐Up Measures

*Social communication, autistic traits, and repetitive behavior*. At 14 months, we included data from the Infant‐Toddler Checklist (ITC; Wetherby and Prizant 2002), which is a 24‐item parent‐rated questionnaire, used to identify children with any type of socio‐communicative delay. Higher scores indicate a higher degree of socio‐communicative abilities. Items include, for example, questions on whether the parent knows when the child is happy or sad, and whether the child lets the parent know when they need help reaching an object. We used the total score as a measure of socio‐communicative abilities at 14 months of age. At 24 months, we included data from the Quantitative Checklist for Autism in Toddlers (Q‐CHAT; Allison et al. 2008), which is a normally distributed quantitative measure of autistic traits and consists of 25 parent‐rated items scored on a 5‐point scale (0–4). The scores from all items are summed to obtain a total score, where higher scores indicate more autistic traits. In addition, we included the Repetitive Behaviors Questionnaire (RBQ; Leekam et al. 2007) at 24 months of age. This is a 20‐item parent‐rated questionnaire that assesses restricted, repetitive, and sensory behaviors. A total mean score (scale 1.00–3.00) is calculated for each child by adding the responses for each item completed in the questionnaire and dividing by the number of questions completed by the parent. Higher scores indicate higher levels of repetitive behaviors.

*Language development*. At 14 months of age, we included data from the MacArthur Communicative Development Inventory, words and gestures form, Swedish version (CDI; Fenson et al. 1993), which is a parent‐rated questionnaire on language development. As a measure of receptive vocabulary at 14 months, we used the total number of words (out of 382 words) that the infant could understand (but not necessarily produce). From the 24‐month assessment, we included data from the CDI words and sentences form. We used the vocabulary checklist score as a measure of expressive vocabulary (i.e., the total number of words the child can produce).

*Hyperactivity*. At 24 months, we included data from the Strengths and Difficulties Questionnaire (SDQ, Goodman 1997). This is a parent‐rated questionnaire consisting of 25 items divided into five categories: emotional problems, conduct problems, hyperactivity, peer problems, and prosocial behavior. Here, we used the total score from the hyperactivity subscale.

There were no statistically significant differences between twin 1 and twin 2 regarding ITC total score (*t*(624) = –0.427, *p* = 0.670), receptive vocabulary (*t*(626) = 0.247, *p* = 0.805), Q‐CHAT total score (*t*(545) = 676, *p* = 0.499), expressive vocabulary (*t*(536) = –0.105, *p* = 0.917), or hyperactivity (*t*(547.600) = –1.959, *p* = 0.051).

### 2.5 Statistical Analyses

First, zero‐order correlations were calculated for all background variables and the sleep, settle, and crying variables at 2 months, in order to get an overview of potential associations. Because we had multiple independent variables and multiple dependent variables, we ran a multivariate multiple regression (Alexopoulos 2010), using all background variables as predictors and number of wakeups per night, settle ability, and crying duration as outcomes. Listwise deletion was used to handle missing data. As all variables were included in the same model, and all hypotheses were preregistered (https://osf.io/865wn/), we did not implement a correction of the *p* value. The same approach was taken in order to analyze the association between sleep, settle, and crying measures (which were entered as predictors) and later measures at 14 and 24 months (which were entered as outcomes). In this model, we also included age and sex as predictors, as well as any background variables that showed a statistically significant association with sleep, settle, and crying behaviors.

## 3 Results

Descriptive statistics of the sleep, settle, and crying measures at 2 months, as well as all follow‐up measures, are presented in Table 2 (see Supporting Information S2 for distributional plots of all variables).

**TABLE 2:** Descriptive statistics of sleep, settle, and crying measures at 2 months, as well as follow‐up measures at 14 and 24 months.

	*N*	Mean (SD) [Min; Max]			Skewness	Kurtosis
		Total	Males	Females		
2 Months						
Wakeups per night (*n*)	353	2.23 (1.11) [0; 10]	2.36 (1.24) [0; 10]	2.11 (0.98) [0; 6]	1.32	6.55
Time until settled (minutes)^a^	358	20.56 (14.62) [0; 111.67]	19.30 (14.30) [0; 80]	21.68 (14.85) [0; 111.67]	1.69	4.81
Crying duration (minutes)^a^	357	23.42 (24.25) [0; 170]	21.63 (22.21) [0; 160]	24.99 (25.87) [0; 170]	2.52	9.33
14 Months						
ITC	317	33.75 (7.23) [9; 55]	32.85 (7.11) [9; 55]	34.52 (7.27) [11; 50]	−0.40	0.31
CDI, receptive vocabulary	318	85.77 (66.20) [0; 382]	79.78 (61.64) [1; 381]	90.85 (69.61) [0; 382]	1.50	3.32
24 Months						
Q‐CHAT	281	25.81 (8.27) [8; 64]	27.65 (8.95) [11; 64]	24.23 (7.31) [8; 43]	0.88	2.05
RBQ	279	1.38 (0.27) [1.00; 2.35]	1.41 (0.28) [1.00; 2.35]	1.36 (0.27) [1.00; 2.15]	1.02	0.62
CDI, vocabulary	277	175.46 (155.47) [0; 649]	135.16 (130.67) [0; 470]	210.59 (166.88) [3; 649]	0.95	<–0.01
SDQ, hyperactivity	292	3.04 (2.10) [0; 10]	3.47 (2.17) [0; 10]	2.69 (1.98) [0; 10]	0.86	0.65

Abbreviations: CDI, communicative development inventory; ITC, infant‐toddler checklist; Q‐CHAT, quantitative checklist for autism in toddlers; RBQ, repetitive behaviors questionnaire; SDQ, strengths and difficulties questionnaire.

^a^The average per time interval (day, evening, night).

As shown in Table 3, statistically significant positive associations were found between number of wakeups per night and both sex (with males waking up more frequently than females; *p* = 0.040) and crying duration (*p* < 0.001). Negative associations were found between wakeups per night and both corrected age at the 2‐month assessment (*p* < 0.001) and gestational age (*p* = 0.007). Time until settled was negatively associated with family income (*p* = 0.021) and positively associated with crying duration (*p* < 0.001). Crying duration showed statistically significant negative associations with gestational age (*p* = 0.002), age (*p* < 0.001), and family income (*p* = 0.036).

**TABLE 3:** Zero‐order correlations between background variables and sleep, settle, and crying measures.

	1	2	3	4	5	6	7	8	9	10	11	12
1 Sex	1											
2 Corrected age	0.016	1										
3 Birthweight	0.111^*^	0.219^**^	1									
4 Gestational age	0.020	0.487^**^	0.542^**^	1								
5 Family income	−0.005	0.075	−0.016	0.070	1							
6 Maternal age	0.007	0.090	0.109^*^	0.129^*^	0.227^**^	1						
7 Paternal age	0.027	0.122^*^	0.147^**^	0.185^**^	0.244^**^	0.690^**^	1					
8 Geographical region	−0.025	0.060	0.000	0.068	−0.004	−0.027	0.019	1				
9 Daylight exposure	−0.064	0.033	−0.036	0.038	0.084	−0.035	−0.006	0.013	1			
10 Wakeups per night	0.109^*^	−0.236^**^	0.037	−0.144^**^	−0.030	0.058	0.030	−0.025	0.014	1		
11 Time until settled	−0.082	−0.079	−0.043	−0.052	−0.123^*^	−0.004	0.018	−0.002	−0.077	0.093	1	
12 Crying duration	−0.069	−0.185^**^	−0.128^*^	−0.160^*^	−0.111^*^	−0.078	−0.034	−0.029	0.092	0.182^**^	0.400^**^	1

* *p* < 0.05.

** *p* < 0.001.

As described in the Section 2, all background variables were entered as predictors in a multivariate multiple regression (*N* = 325), and the sleep, settle, and crying variables were entered as outcomes. While some of the predictors showed a statistically significant association with each other, the VIF values for the predictors ranged from 1.02 to 2.04, suggesting that they can be entered into the same model. Corrected age, family income, and daylight exposure were statistically significant predictors of the combined outcomes (Table 4). No significant effects were found regarding sex, birthweight, gestational age, geographical location, maternal age, or paternal age. Test of between‐subjects effects showed that corrected age (27–99 days) predicted number of wakeups per night (*β* = –0.212, *p* < 0.001, partial eta squared = 0.040) and crying duration (*β* = –0.154, *p* = 0.012, partial eta squared = 0.020), but not settle ability (*p* = 0.290). Family income predicted settle ability (*β* = –0.147, *p* = 0.011, partial eta squared = 0.020) and crying duration (*β* = –0.128, *p* = 0.018, partial eta squared = 0.018), but not number of wakeups per night (*p* = 0.349). No significant associations were found between daylight exposure and wakeups per night (*p* = 0.972), settle ability (*p* = 0.081), or crying duration (*p* = 0.075) separately. See Table S1 for full details.

**TABLE 4:** Multivariate multiple regression with background variables as predictors and sleep, settle, and crying variables as outcomes.

	Pillai's trace	F	*p* value	Partial eta squared
Sex	0.017	1.803	0.147	0.017
Corrected age	0.056	6.172	<0.001^*^	0.056
Birthweight	0.015	1.553	0.201	0.015
Gestational age	0.003	0.341	0.796	0.003
Geographical region	0.003	0.326	0.807	0.003
Family income	0.029	3.117	0.026^*^	0.029
Maternal age	0.011	1.188	0.314	0.011
Paternal age	0.010	1.017	0.385	0.010
Daylight exposure	0.031	3.327	0.020^*^	0.031

* *p* < 0.05.

Zero‐order correlations between sleep, settle, and crying variables and later outcomes showed a statistically significant positive association between settle ability at 2 months and receptive vocabulary at 14 months (*p* = 0.035, Table 5).

**TABLE 5:** Zero‐order correlations between sleep, settle, and crying variables and follow‐up measures.

	1	2	3	4	5	6	7	8	9
1 Wakeups per night	1								
2 Time until settled	0.093	1							
3 Crying duration	0.182^**^	0.400^**^	1						
4 ITC (14 m)	0.016	−0.050	−0.056	1					
5 CDI (14 m)	−0.075	0.119^*^	0.001	0.481^**^	1				
6 Q‐CHAT (24 m)	0.021	0.045	0.005	−0.348^**^	−0.124^*^	1			
7 RBQ (24 m)	−0.016	0.075	−0.033	0.051	0.082	0.516^**^	1		
8 SDQ (24 m)	0.001	0.046	0.048	−0.206^**^	−0.025	0.258^**^	0.130^*^	1	
9 CDI (24 m)	−0.014	0.035	0.048	0.449^**^	0.426^**^	−0.287^**^	−0.013	−0.116	1

Abbreviations: CDI, communicative development inventory; ITC, infant‐toddler checklist; Q‐CHAT, quantitative checklist for autism in toddlers; RBQ, repetitive behaviors questionnaire; SDQ, strengths and difficulties questionnaire.

* *p* < 0.05.

** *p* < 0.001.

In line with the planned analyses, the sleep, settle, and crying variables, along with sex, corrected age, and family income, were then entered as predictors in a multivariate multiple regression (*N* = 243), and all follow‐up measures were entered as outcomes. While some of the predictors showed a statistically significant association with each other, the VIF values for the predictors ranged from 1.00 to 1.20, suggesting that they can be entered into the same model. As can be seen in Table 6, sex was a significant predictor of later outcomes, but no other predictor reached statistical significance. Between‐subjects tests showed that boys had higher scores than girls on the Q‐CHAT (*β* = 0.533, *p* < 0.001, partial eta squared = 0.068), RBQ (*β* = 0.265, *p* = 0.039, partial eta squared = 0.018), and hyperactivity (*β* = 0.449, *p* < 0.001, partial eta squared = 0.048), while the opposite pattern was found for vocabulary (*β* = –0.521, *p* < 0.001, partial eta squared = 0.067). Full details are reported in Table S2. In light of the correlation between outcome variables, we also tested the same associations using a rank‐reduced regression technique. This is due to the fact that intercorrelated outcomes cannot be inferred as if they are independent, and thus, the method of coefficient estimation of the models should be modified accordingly (Izenman 1975). Therefore, we constrained (i.e., reduced) the rank of the matrix containing those betas. The conclusions did not change (see Supporting Information S3 for full details).

**TABLE 6:** Multivariate multiple regression with sleep, settle, and crying variables, as well as age, sex, and family income as predictors, and follow‐up measurements as outcomes.

	Pillai's trace	F	*p* value	Partial eta squared
Sex	0.135	5.986	<0.001^*^	0.135
Corrected age	0.006	0.219	0.970	0.006
Family income	0.010	0.403	0.877	0.010
Wakeups per night (*n*)	0.010	0.382	0.890	0.010
Time until settled (minutes)	0.045	1.841	0.092	0.045
Crying duration (minutes)	0.020	0.810	0.563	0.020

* *p *< 0.001.

In accordance with the pre‐registered plan, we also tested the association between later outcomes and the settle and crying measures separately for daytime, evening, and nighttime. These six variables, together with corrected age, sex, and family income, were entered as predictors in a multivariate multiple regression (*N* = 235), and all follow‐up measures were entered as outcomes. None of the settle, and crying variables were significant predictors of the combined outcomes (details are reported in Table S3).

## 4 Discussion

The aim of this study was to examine the associations between a range of background measures and sleep, settle, and crying behaviors at 2 months of age. In addition, we investigated whether these behaviors at 2 months predict later abilities and difficulties at 14 and 24 months of age.

Corrected age at the 2‐month assessment, which in our study ranged from 27 to 99 days, was a significant predictor of number of wakeups per night and crying duration. This association was negative, meaning that older infants woke up less frequently and cried for shorter durations. This result was expected, and is in line with earlier research describing the development of sleep and crying in young infants (Figueiredo et al. 2016; McGlaughlin and Grayson 2001; St James‐Roberts and Plewis 1996). While approximate daylight exposure was a significant predictor of sleep, settle, and crying behaviors combined in the multivariate regression, the associations between daylight exposure and each measure separately did not reach statistical significance, suggesting that natural daylight exposure may not play an important role for any of these specific sleep and settle behaviors in early infancy. An earlier study found a positive association between infant sleep quality and light exposure during the day (Harrison 2004), but that study measured light conditions in general, regardless of it being daylight or artificial light. Another study found that nighttime sleep duration was positively associated with regular light‐off times in the home (Iwata et al. 2017). Taken together, these results suggest that light conditions are potentially important for early sleep, and that this encompasses light conditions in general, not merely daylight exposure. It is also noteworthy that our operationalization of daylight was based on the number of hours the sun was above the horizon in the area at the time the parents filled in the questionnaires, but it did not take weather (e.g., cloud cover) or time spent outdoors into account. Still, it is notable that our sample had a variation ranging from 3 to 24 h of daylight exposure.

Higher family income was associated with shorter duration of crying and less time until the infant settled. It is possible that this association is due to different parental practices. One study of 3–4‐month‐old infants found that infants in upper SES families engaged in more self‐play, vocalized less, and fussed less, than did infants in the middle and lower SES families (Fouts et al. 2007). Upper SES families also provided more verbal affection and soothing responses to their fussing and crying infants than did the middle and lower SES families. While this may be true also in our sample, we did not measure parental soothing practices, and further studies are needed in order to understand the link between family income and crying/settle behaviors. It is also possible that the link between SES and crying can partly be explained by genetic effects. Socio‐economic status involves genetically influenced traits (Abdellaoui et al. 2025), and we found a high genetic influence on crying duration at two months of age in a study using partly the same sample as in the current study (heritability estimates of 0.44–0.48; Viktorsson et al. 2025). It is worth noting that the mean family income in our sample was 60–70K, which is in line with the national median salary in Sweden (35.6K per person, www.scb.se), and that only 5% (18 families) of our sample reported that they have had difficulties paying for current expenses during the last 12 months. Thus, in general the sample seems to reflect the Swedish population, meaning that the standard of living is quite high and there are social welfare systems available to those in need. Future studies should explore the link between SES and regulatory difficulties in more diverse and vulnerable samples, where the need for support and help might be higher.

In line with previous research, at follow up males exhibited more autistic traits, more repetitive behavior, and more hyperactivity, while females had a larger vocabulary (e.g., Ellis and He 2014; Mandy et al. 2018; Ozcaliskan and Goldin‐Meadow 2010). These traits and abilities were not significantly associated with number of wakeups per night, crying duration, or time until the infant settled at 2 months of age, which might suggest that the sleep and settle behaviors measured in this study are not yet important for later development at this early age. As described in the introduction, an earlier study found that infants later diagnosed with autism slept less within a 24‐h period than typically developing infants across the first 6 months of life (Foster et al. 2023), but there were no group differences regarding nighttime sleep duration, and it was found that the 24‐h sleep duration was not related to autistic traits at 24 months. Another study found that infants with later autism exhibited shorter sleep duration, more night awakenings and more sleep onset problems than infants without later autism (Begum‐Ali et al. 2023). However, the association was only found at 14 months, not at 5 or 10 months, suggesting that the link between early sleep difficulties and later autism may not be present during the first months of life. It is also possible that these potential links are not present in the general population, as the previous studies focused on children with elevated likelihood of autism and later autism diagnosis, while the present study is based on a general population sample and focus on parent‐rated autistic traits. In addition, it is important to note that earlier studies measured sleep duration, while the current study focus on night awakenings and settle time, in addition to crying duration.

In both typical and atypical development, the first months of life is a period of significant developmental changes, both in terms of behaviors and their underlying etiology. A recent twin study using partly the same sample as in the current study found that there are only modest shared genetic and environmental influences across 2 and 5 months of age with regards to sleep, settle, and crying behaviors (Viktorsson et al. 2025). These significant changes in etiology during the first months of life might also partly explain why we do not find the same links to later behaviors as earlier studies of older infants. Future research should test the association between sleep and settle measures and later traits at multiple timepoints, in order to pinpoint potential time windows where sleep and settle behaviors are significant for later development, and therefore could be targets of early interventions.

In this study we relied on parent‐reported questionnaires, which may not reflect the sleep and settle behaviors accurately. While a high correlation between parent‐reports and objectively measured sleep duration was found in a sample of 3–4‐year‐olds (Sekine et al. 2002), other studies have found significant differences between mother‐reported and actigraphy‐obtained sleep durations. For example, one study found that on average, mothers tended to overestimate infants’ total 24‐h sleep duration by ∼0.5–1.5 h/day when compared to actigraphy at 4–6 months of age (Adams et al. 2019). Another study found that mothers generally reported somewhat less wakeups per night than the actigraphy‐obtained data for 2‐year‐olds (Simard et al. 2013). In the current study, the parents filled in the questionnaire online, and while the large variation in reported sleep and settle behaviors may reflect true individual differences, it might also partially reflect different interpretations of the questions. For example, the questionnaire does not specify the precise onset of the “time until settled” period, and parents may therefore have differed in whether they considered it to begin with the bedtime routine or once the infant was placed in bed. At the same time, the Swedish term for settle, *komma till ro*, is generally understood as the process of calming down prior to sleep, after being placed in bed. In order to minimize the risk of different interpretations, future studies should either obtain the answers by parents through interviews or implement clarifications in the questionnaire. In addition to sleep and settle behaviors, all outcome measures relied on parent report. While parent‐rated questionnaires are widely used and provide valuable insights, they are vulnerable to bias. Factors such as parental stress or mental health may influence perceptions of both early sleep and later behavioral difficulties, leading to associations that reflect shared reporting effects rather than true covariation. Future research should incorporate objective measures (e.g., actigraphy) and observations or teacher ratings of child behavior, in order to strengthen the validity and reduce potential bias.

It is important to note that we used listwise deletion in all analyses, and due to attrition, the sample size was considerably smaller for follow‐up measures than for the 2‐month assessment. Therefore, many participants were excluded from the analysis where follow‐up measures were entered as outcomes, which reduced statistical power.

While caring for twins might pose higher demands for the parents than caring for a single infant, a comparison study of twins (using partly the same sample as the current study) and singletons at 5 months of age found no differences regarding settle and crying behaviors (Viktorsson et al. 2024). There was a significant difference in the number of wakeups, but the twins woke up less frequently than the singletons, and the number of night awakenings in the twin sample was comparable to what has been found in other studies of singletons (e.g., Paavonen et al. 2020). Therefore, despite the unique demands of twin parents, it is reasonable to believe that these findings are generalizable to singletons.

## 5 Conclusions

Sleep, settle, and crying behaviors reflect important developmental processes in early infancy, but little is known about what factors may influence these behaviors and how they relate to later development. In a sample of more than 300 infants, we found that corrected age significantly predicted both number of wakeups per night and crying duration, while family income predicted both crying duration and the time it took for the infant to settle. These behaviors did not predict parent‐reported language comprehension or socio‐communicative abilities at 14 months, nor did they predict vocabulary, autistic traits, or hyperactivity at 24 months. This suggests that sleep and settle behaviors in the first couple of months, a time of significant developmental change, are not indicative of abilities and difficulties in toddlerhood in a general population sample.

## Author Contributions

The hypotheses and goals of this study were conceptualized by C.V. and T.F.‐Y. Data were analyzed by C.V. and I.H. C.V. and A.J. drafted the manuscript, and all of the authors reviewed, edited, and approved the final manuscript for submission.

## Funding

This research was funded by the Knut and Alice Wallenberg Foundation (grant number 2019.0167), the Swedish Research Council (grant number 2018‐06232_VR), Stiftelsen Sunnerdahls Handikappsfond (grant number D7/22), and the Innovative Medicines Initiative 2 Joint Undertaking (grant number 777394). This Joint Undertaking receives support from the European Union's Horizon 2020 Research and Innovation Programme and EFPIA and AUTISM SPEAKS, Autistica, SFARI. The work was also supported by funds from the European Commission (H2020 project CANDY; grant number 847818). Any views expressed are those of the author(s) and not necessarily those of the funders. The funders had no role in the design of the study; in the collection, analyses, or interpretation of data, in the writing of the manuscript, or in the decision to publish the results.

## Ethics Statement

Informed consent was obtained from all caregivers. The study was approved by the regional ethics board in Stockholm (2020‐03226) and was conducted in accordance with the Declaration of Helsinki.

## Conflicts of Interest

The authors declare no conflicts of interest.

## Supporting information




**Supporting File 1**: desc70126‐sup‐0001‐SuppMat.docx

## Data Availability

The analyses presented here were preregistered (https://osf.io/865wn/). The data and code necessary to reproduce the analyses presented here are not publicly accessible, but will be made available upon reasonable request to the corresponding author. Note that sharing of pseudonymized personal data will require a data sharing agreement, according to Swedish and EU law.
