# Supplementary material for: How Are Sleep, Settle, and Crying Behaviors in 2‐Month‐Olds Related to Concurrent Family Factors and Later Development?
Source: Dev Sci. 2026 Jan 5;29(2):e70126. doi: 10.1111/desc.70126 (PMC12770087; doi:10.1111/desc.70126)
Supplement: Supplementary file 1 — Supporting File 1: desc70126‐sup‐0001‐SuppMat.docx [file DESC-29-e70126-s001.docx]

Supplementary information

**How are sleep, settle, and crying behaviors in 2-month-olds related to concurrent family factors and later development?**

**Supplementary Information S1**. Distributional plots of all background variables (for twin 1).


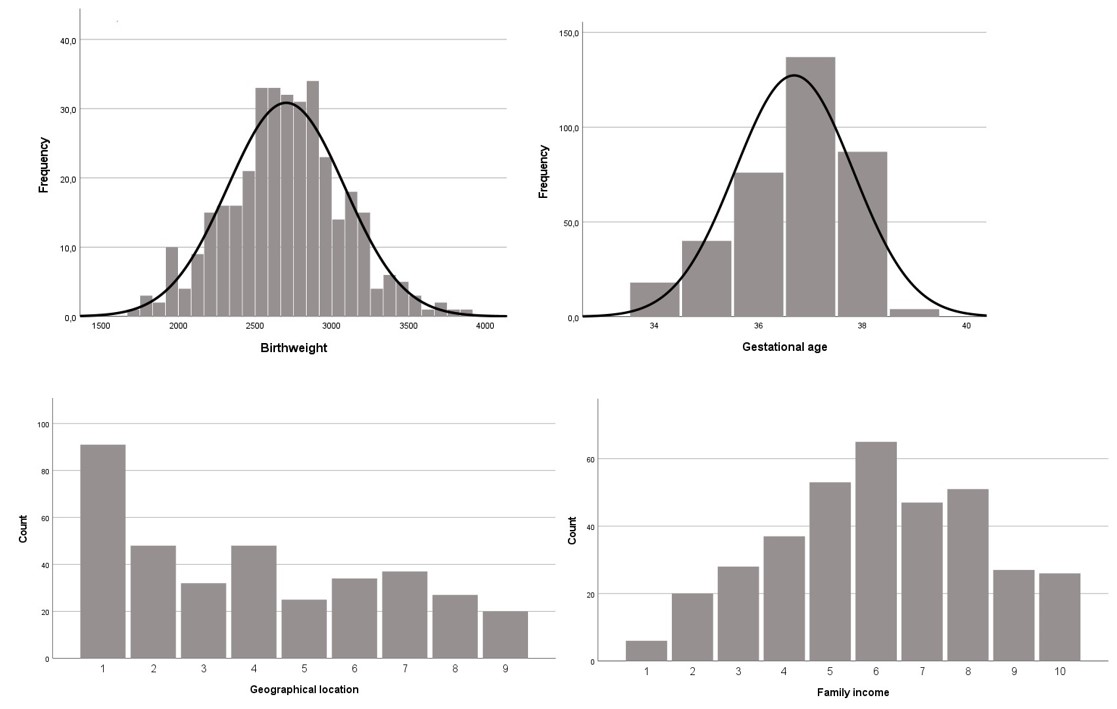


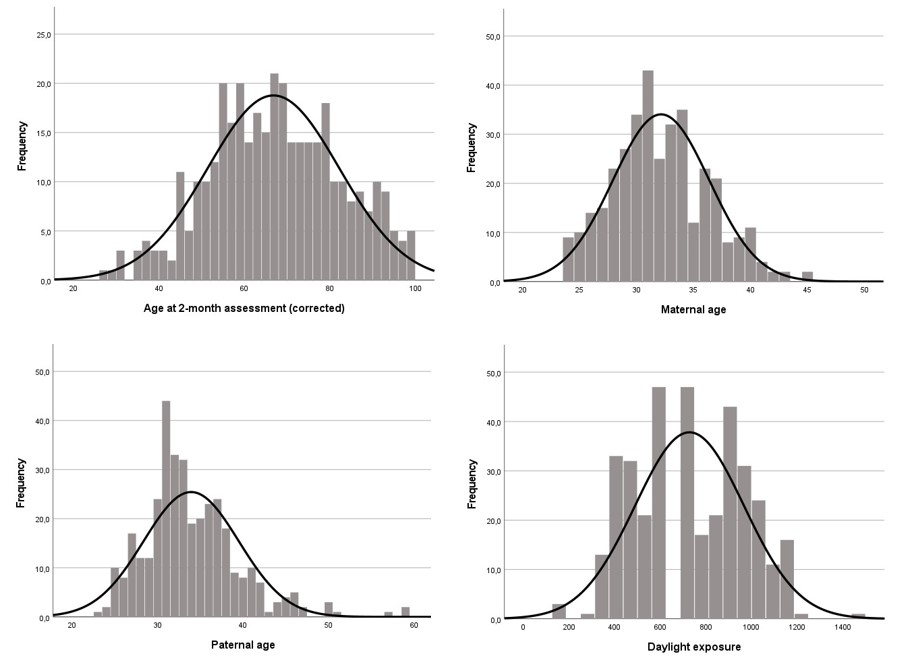


**Supplementary Information S2**. Distributional plots of all sleep, settle, and crying variables, as well as follow-up measures (for twin 1).


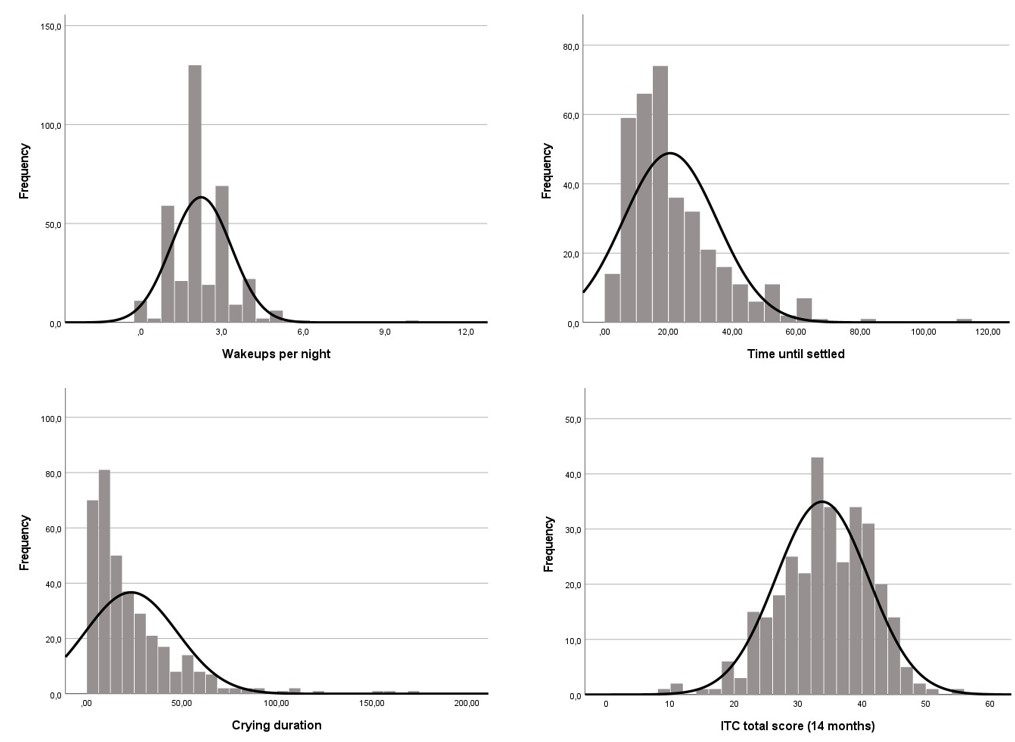


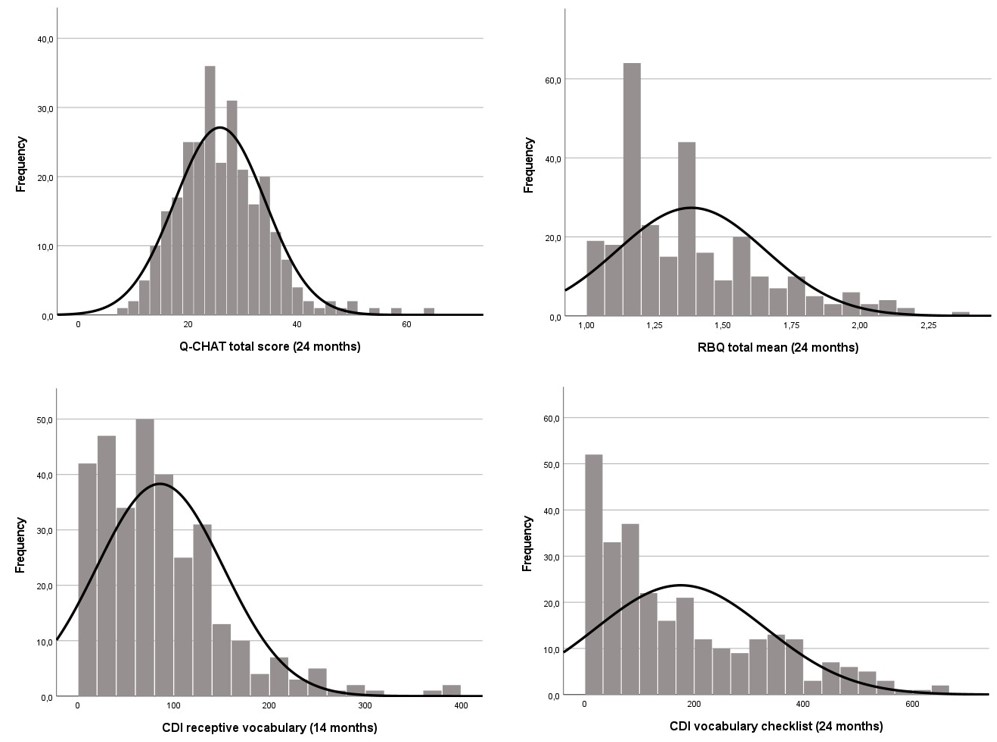


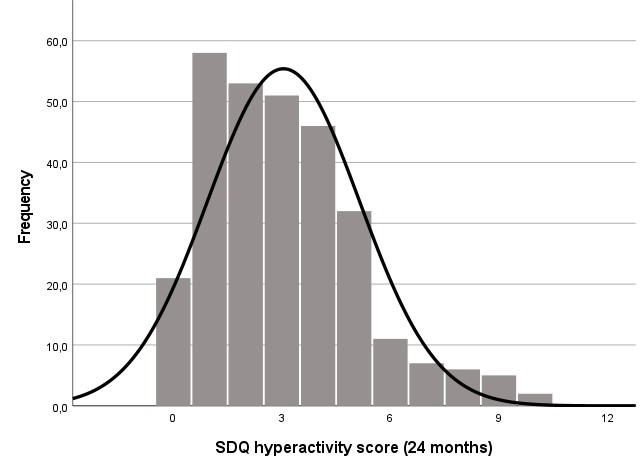


**Supplementary Information S3**. Applying rank-reduced regression technique.

First, we looked at the rank trace plot of the beta matrix of each multivariate model (i.e., background variables vs. sleep/settle variables, and sleep/settle variables vs. later outcomes) to decide on the rank number to be used. For the first model (background variables vs. sleep/settle variables), the rank number was 3, which was equal to the original model, meaning that no rank-reduced technique was applied. For the second model (sleep/settle variables vs. later outcomes), the rank number was 4 or 5 (see figure below). We decided to fit a rank-reduced model with a rank of 5 (in order to be more conservative). The results were very similar to the ones we got from the original model (see table below), meaning that they do not change the conclusions from the classical multivariate approach.


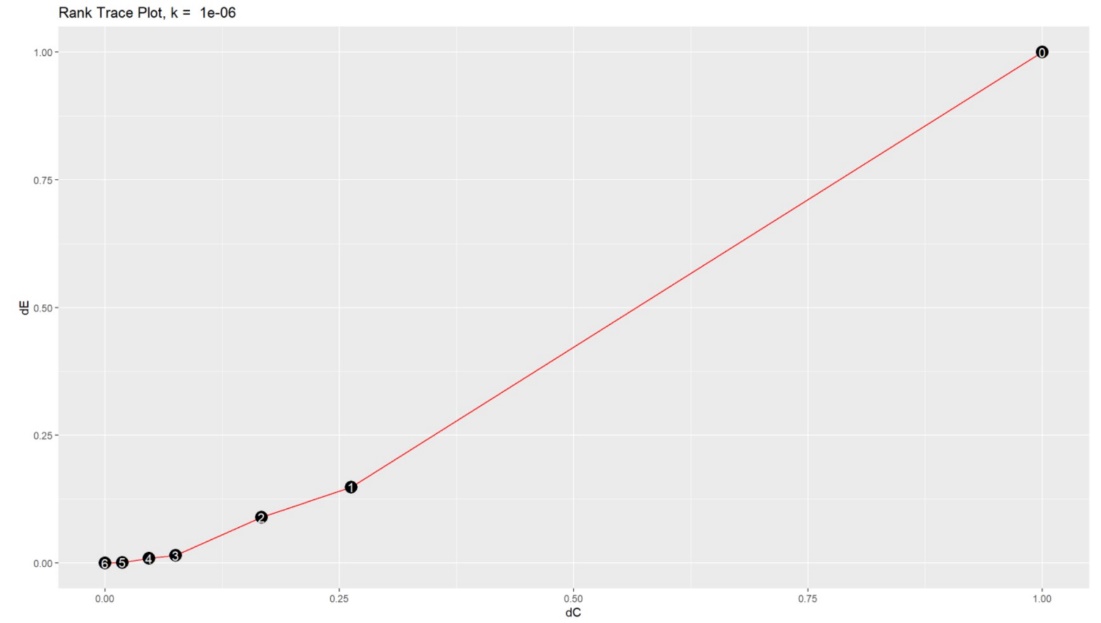


|  |  | **t-statistic** | **p-value** | **Standardized β** |
| --- | --- | --- | --- | --- |
| Sex | ITC (14 months) | 1.432 | 0.153 | -0.201 |
|  | Receptive vocabulary (14 months) | -0.877 | 0.382 | -0.102 |
|  | Q-CHAT (24 months) | 4.208 | <.001 | 0.533 |
|  | RBQ (24 months) | 2.111 | 0.036 | 0.266 |
|  | Vocabulary (24 months) | -4.177 | <.001 | -0.522 |
|  | SDQ (24 months) | 3.486 | 0.001 | 0.449 |
| Age | ITC (14 months) | 0.927 | 0.355 | 0.062 |
|  | Receptive vocabulary (14 months) | 0.320 | 0.749 | 0.020 |
|  | Q-CHAT (24 months) | -0.653 | 0.514 | -0.043 |
|  | RBQ (24 months) | 0.184 | 0.854 | 0.012 |
|  | Vocabulary (24 months) | 0.287 | 0.774 | 0.019 |
|  | SDQ (24 months) | -0.473 | 0.637 | -0.032 |
| Family income | ITC (14 months) | -0.260 | 0.795 | -0.017 |
|  | Receptive vocabulary (14 months) | -1.124 | 0.262 | -0.066 |
|  | Q-CHAT (24 months) | 0.133 | 0.894 | 0.008 |
|  | RBQ (24 months) | -0.170 | 0.865 | -0.011 |
|  | Vocabulary (24 months) | 0.412 | 0.680 | 0.026 |
|  | SDQ (24 months) | 0.264 | 0.792 | 0.017 |
| Wakeups per night (n) | ITC (14 months) | 0.526 | 0.599 | 0.037 |
|  | Receptive vocabulary (14 months) | -0.771 | 0.442 | -0.049 |
|  | Q-CHAT (24 months) | -0.297 | 0.766 | -0.021 |
|  | RBQ (24 months) | -0.750 | 0.454 | -0.052 |
|  | Vocabulary (24 months) | -0.150 | 0.881 | -0.010 |
|  | SDQ (24 months) | -0.675 | 0.500 | -0.047 |
| Time until settled | ITC (14 months) | -1.364 | 0.174 | -0.092 |
| (minutes) | Receptive vocabulary (14 months) | 1.520 | 0.130 | 0.093 |
|  | Q-CHAT (24 months) | 1.234 | 0.219 | 0.082 |
|  | RBQ (24 months) | 1.466 | 0.144 | 0.097 |
|  | Vocabulary (24 months) | -0.044 | 0.965 | -0.003 |
|  | SDQ (24 months) | 0.825 | 0.410 | 0.056 |
| Crying duration | ITC (14 months) | -0.460 | 0.646 | -0.034 |
| (minutes) | Receptive vocabulary (14 months) | -1.235 | 0.218 | -0.084 |
|  | Q-CHAT (24 months) | -0.671 | 0.503 | -0.050 |
|  | RBQ (24 months) | -1.072 | 0.285 | -0.079 |
|  | Vocabulary (24 months) | 0.355 | 0.723 | 0.026 |
|  | SDQ (24 months) | 1.119 | 0.264 | 0.084 |

Tables and figures

**Table S1**. Multivariate multiple regression with background variables as predictors, and sleep, settle, and crying variables as outcomes.

|  |  | **F** | **p-value** | **Partial eta squared** | **Standardized β** |
| --- | --- | --- | --- | --- | --- |
| Sex | Wakeups per night | 1.565 | 0.212 | 0.005 | 0.122 |
|  | Time until settled | 2.354 | 0.126 | 0.007 | -0.167 |
|  | Crying duration | 2.637 | 0.105 | 0.008 | -0.174 |
| Age | Wakeups per night | 13.229 | <.001* | 0.040 | -0.212 |
|  | Time until settled | 1.096 | 0.296 | 0.003 | -0.068 |
|  | Crying duration | 6.507 | 0.011* | 0.020 | -0.154 |
| Birthweight | Wakeups per night | 2.634 | 0.106 | 0.008 | 0.095 |
|  | Time until settled | 0.264 | 0.607 | 0.001 | -0.032 |
|  | Crying duration | 1.980 | 0.160 | 0.006 | -0.090 |
| Gestational age | Wakeups per night | 1.122 | 0.290 | 0.004 | -0.068 |
|  | Time until settled | 0.002 | 0.966 | 0.000 | 0.001 |
|  | Crying duration | 0.001 | 0.977 | 0.000 | 0.004 |
| Geographical location | Wakeups per night | 0.329 | 0.567 | 0.001 | -0.037 |
|  | Time until settled | 0.207 | 0.650 | 0.001 | 0.015 |
|  | Crying duration | 0.047 | 0.828 | 0.000 | -0.041 |
| Family income | Wakeups per night | 0.558 | 0.456 | 0.002 | -0.049 |
|  | Time until settled | 6.797 | 0.010* | 0.021 | -0.147 |
|  | Crying duration | 5.437 | 0.020* | 0.017 | -0.128 |
| Maternal age | Wakeups per night | 1.175 | 0.279 | 0.004 | 0.071 |
|  | Time until settled | 0.033 | 0.856 | 0.000 | -0.011 |
|  | Crying duration | 2.173 | 0.141 | 0.007 | -0.110 |
| Paternal age | Wakeups per night | 0.052 | 0.820 | 0.000 | 0.020 |
|  | Time until settled | 0.892 | 0.346 | 0.003 | 0.070 |
|  | Crying duration | 2.829 | 0.094 | 0.009 | 0.125 |
| Daylight exposure | Wakeups per night | 0.001 | 0.971 | 0.000 | 0.002 |
|  | Time until settled | 3.083 | 0.080 | 0.010 | -0.098 |
|  | Crying duration | 3.160 | 0.076 | 0.010 | 0.094 |

* = p <.05

**Table S2**. Multivariate multiple regression with sleep, settle, and crying variables, as well as age, sex, and family income as predictors, and follow-up measurements as outcomes.

|  |  | **F** | **p-value** | **Partial eta squared** | **Standardized β** |
| --- | --- | --- | --- | --- | --- |
| Sex | ITC (14 months) | 2.358 | 0.126 | 0.010 | -0.200 |
|  | Receptive vocabulary (14 months) | 0.742 | 0.390 | 0.003 | -0.102 |
|  | Q-CHAT (24 months) | 17.247 | <.001* | 0.068 | 0.533 |
|  | RBQ (24 months) | 4.308 | 0.039* | 0.018 | 0.265 |
|  | Vocabulary (24 months) | 16.914 | <.001* | 0.067 | -0.521 |
|  | SDQ (24 months) | 11.816 | <.001* | 0.048 | 0.449 |
| Age | ITC (14 months) | 0.724 | 0.396 | 0.003 | 0.058 |
|  | Receptive vocabulary (14 months) | 0.077 | 0.782 | 0.000 | 0.017 |
|  | Q-CHAT (24 months) | 0.534 | 0.466 | 0.002 | -0.049 |
|  | RBQ (24 months) | 0.061 | 0.805 | 0.000 | 0.017 |
|  | Vocabulary (24 months) | 0.052 | 0.819 | 0.000 | 0.015 |
|  | SDQ (24 months) | 0.250 | 0.617 | 0.001 | -0.034 |
| Family income | ITC (14 months) | 0.069 | 0.793 | 0.000 | -0.017 |
|  | Receptive vocabulary (14 months) | 1.235 | 0.268 | 0.005 | -0.066 |
|  | Q-CHAT (24 months) | 0.015 | 0.902 | 0.000 | 0.008 |
|  | RBQ (24 months) | 0.026 | 0.872 | 0.000 | -0.010 |
|  | Vocabulary (24 months) | 0.161 | 0.689 | 0.001 | 0.026 |
|  | SDQ (24 months) | 0.066 | 0.798 | 0.000 | 0.017 |
| Wakeups per night (n) | ITC (14 months) | 0.013 | 0.908 | 0.000 | 0.031 |
|  | Receptive vocabulary (14 months) | 0.899 | 0.344 | 0.004 | -0.052 |
|  | Q-CHAT (24 months) | 0.008 | 0.931 | 0.000 | -0.029 |
|  | RBQ (24 months) | 0.329 | 0.567 | 0.001 | -0.045 |
|  | Vocabulary (24 months) | 0.487 | 0.486 | 0.002 | -0.015 |
|  | SDQ (24 months) | 0.119 | 0.730 | 0.000 | -0.051 |
| Time until settled | ITC (14 months) | 1.703 | 0.193 | 0.007 | -0.095 |
| (minutes) | Receptive vocabulary (14 months) | 2.609 | 0.108 | 0.011 | 0.092 |
|  | Q-CHAT (24 months) | 0.947 | 0.331 | 0.004 | 0.079 |
|  | RBQ (24 months) | 2.027 | 0.156 | 0.008 | 0.100 |
|  | Vocabulary (24 months) | 0.003 | 0.953 | 0.000 | -0.005 |
|  | SDQ (24 months) | 0.400 | 0.528 | 0.002 | 0.055 |
| Crying duration | ITC (14 months) | 0.194 | 0.660 | 0.001 | -0.035 |
| (minutes) | Receptive vocabulary (14 months) | 1.284 | 0.258 | 0.005 | -0.084 |
|  | Q-CHAT (24 months) | 0.680 | 0.411 | 0.003 | -0.050 |
|  | RBQ (24 months) | 1.392 | 0.239 | 0.006 | -0.078 |
|  | Vocabulary (24 months) | 0.186 | 0.667 | 0.001 | 0.025 |
|  | SDQ (24 months) | 0.870 | 0.352 | 0.004 | 0.084 |

**Table S3**. Multivariate multiple regression with age, sex, family income, and settle and crying measures (at different times of the day) as predictors, and follow-up measures as outcomes.

|  | **Pillai’s Trace** | **F** | **p-value** | **Partial eta squared** |
| --- | --- | --- | --- | --- |
| Sex | 0.127 | 5.345 | <.001* | 0.127 |
| Age | 0.007 | 0.267 | 0.952 | 0.007 |
| Family income | 0.009 | 0.531 | 0.909 | 0.009 |
| Time until settled (daytime) | 0.036 | 1.369 | 0.228 | 0.036 |
| Time until settled (evening) | 0.024 | 0.883 | 0.508 | 0.024 |
| Time until settled (nighttime) | 0.048 | 1.854 | 0.090 | 0.048 |
| Crying duration (daytime) | 0.012 | 0.433 | 0.856 | 0.012 |
| Crying duration (evening) | 0.010 | 0.363 | 0.902 | 0.010 |
| Crying duration (nighttime) | 0.016 | 0.605 | 0.726 | 0.016 |

* = p <.05
